# The effect of one-night sleep deprivation on cognitive functions in healthy young adults

**DOI:** 10.5935/1984-0063.20200066

**Published:** 2021

**Authors:** Serkan Pekçetin, Gülnur Öztürk, Buse Çetin, Levent Öztürk

**Affiliations:** 1 University of Health Sciences Turkey, Faculty of Gülhane Health Sciences, Department of Occupational Therapy- Ankara, Turkey.; 2 Trakya University, Faculty of Health Sciences, Department of Physical Therapy and Rehabilitation - Edirne, Turkey.; 3 Çorlu Public Hospital, Deparment of Physical Therapy and Rehabilitation - Tekirdağ, Turkey.; 4 Trakya University, Faculty of Medicine, Department of Physiology - Edirne, Turkey.

**Keywords:** Sleep Deprivation, Cognition, Adult

## Abstract

**Introduction:**

The effects of acute sleep deprivation on cognitive function have not been clearly elucidated. The purpose of this study was to evaluate changes in cognitive function in healthy adults after one night of sleep deprivation.

**Material and Methods:**

Twenty-one healthy young adults (aged 18-30 years) underwent assessment of cognitive functions before and after one night of total sleep deprivation and an age- and gender-matched control group was assessed before and after a normal night sleep. Cognitive functions were assessed using the Montreal cognitive assessment (MOCA) and trail making test (TMT) parts A and B.

**Results:**

General linear model repeated measures demonstrated an insigniﬁcant effect for time × group (sleep deprivation) interaction for MOCA, TMT Part A, and TMT Part B scores after one-night sleep deprivation (p>.05 for all).

**Conclusion:**

A single night of sleep deprivation, which can be inevitable in modern society, had no signiﬁcant effect on cognitive performance in healthy adults.

## INTRODUCTION

Sleep is a physiologic need that takes up approximately one-third of a human’s life. Proper daytime functioning depends on an adequate amount of sleep, and sleep quality also has an impact on our everyday occupations^1^. The average individual needs 7 to 8 hours of sleep a day. Curtailment of sleep duration or sleep abnormalities adversely impact health, immunity, mood, quality of life, and cognitive function^2^.

There are certain vocations in which individuals have to cope with the effects of sleep deprivation due to round-the-clock services, such as in the health, security, and military sectors. Shift-workers account for 8% of the working population in Turkey^3^. However, it can be estimated that sleep deprivation is much more common in the general population when we add to these occupations other groups that tend to have inadequate or irregular sleep schedules, such as academicians, students, and people who sacrifice sleep for socializing. A recent study conducted in Turkey showed that 23% of young adults sleep less than 6 hours per night^4^. Therefore, it is important to evaluate the effect of sleep deprivation on cognition in this population.

Acute sleep deprivation (ASD) is prolonged wakefulness for a single period and it may affect several components of an individual’s cognitive functions, including executive functioning, attention, working memory, long-term memory, visual-motor performance, decision-making, and verbal functions. There are two main theories to explain these negative effects. The first is that ASD leads to decreased alertness and attention, which results in cognitive impairments. According to this theory, attentional lapses are the main reason for the decrease in cognitive performance^5^. The second theory is that ASD has a negative impact on the prefrontal cortex. Studies have indicated that ASD leads to a decrement of brain metabolism in the prefrontal cortex^6,7^ as well as loss of functional connectivity^8^.

However, the literature includes contradictory findings regarding the detrimental effects of ASD on cognition. Some studies suggested that ASD has negative effects on cognitive functions^9,11^ while in other studies ASD had no apparent affect cognition in healthy individuals^12,14^. Therefore, the current study was carried out to investigate the effects of one-night sleep deprivation on cognitive function in healthy young adults.

## MATERIAL AND METHODS

The study protocol was approved by the local ethics committee. Written informed consent was obtained from all participants prior to the study.

### Study groups

Forty-two healthy adults aged 18 to 30 years were recruited for the study using word of mouth and flyers. Exclusion criteria were having any mental or physical health problems or sleep disorders, taking any regular medication, and shift working. The included participants were randomly assigned to the sleep deprivation group (n=21; male/female=11/10) or to the age- and gender-matched control group that slept normally (n=21; male/female=11/10). All participants underwent physical examination by a medical doctor (L.O.) and were found to be healthy. The Pittsburgh sleep quality index (PSQI) and Epworth sleepiness scale (ESS) were applied to rule out any sleep problems. Participants with a PSQI score lower than 5 and ESS score lower than 10 were accepted as normal and included in the study. As all 42 participants met the inclusion criteria, none was excluded from the study.

### Sleep deprivation procedure

Cognitive measurements were performed at two time points. Initial assessments were done at 8:00 a.m. on the first day of the study, and the second assessments were done after one night of total sleep deprivation (at 8:00 a.m.). All tests were carried out by the same researcher (G.O.) who was not included in the sleep deprivation protocol. Two researchers accompanied the participants during the sleep deprivation to ensure they remained awake. The participants were only allowed to perform non-strenuous activities such as reading, playing cards, watching TV, or speaking. Caffeinated beverages and alcohol consumption were forbidden during the study period.

A familiarization process was not implemented in this study; therefore, volunteers in the control group were evaluated at 8:00 a.m. of the first study day and second assessment was performed 24 hours later to measure the learning effect with the instruments. No sleep restriction protocol was implemented for participants in the control group.

### Instruments

#### Pittsburgh Sleep Quality Index (PSQI)

The PSQI is a self-report questionnaire that evaluates sleep quality and sleep disturbances within the last month. It consists of 23 items, each of which is scored on a 3-point scale. Higher score indicates worse sleep quality. Sleep quality is evaluated as good in those with an overall score of 5 points or lower. Ağargün et al. (1996)^15^ performed the Turkish validity and reliability study of the PSQI.

#### Epworth Sleepiness Scale (ESS)

The ESS is designed to evaluate excessive daytime sleepiness. The scale asks individuals to rate their probability of falling asleep in 8 different situations on a scale of 0 to 3 points. A total score higher than 10 points is considered excessive daytime sleepiness. Izci et al. (2008)^16^ performed the Turkish validity and reliability study of the ESS.

#### Montreal Cognitive Assessment (MOCA)

The MOCA is designed to measure the cognitive domains of individuals under visuospatial and executive functions. The subtests include alternating trail making, cube copying, clock drawing, naming (lion, rhinoceros, camel), attention (forward and backward digit span, tapping to the letter A, subtraction from 100 by 7s), language (sentence repetition, letter fluency; abstraction: similarities between train and bicycle, watch and ruler), memory (5-minute delayed verbal recall of 5 words), and orientation to time and place. The total score ranges from 0 to 30 points. Higher score indicates better cognition. Selekler et al. (2010)^17^ demonstrated the validity of Turkish version of the MOCA.

#### Trail Making Test (TMT)

The TMT evaluates visual attention, praxis processing speed, and cognitive flexibility. The TMT broadly represents individuals’ executive functions. The test has two parts. In Part A, numbers are randomly distributed on an A4 paper and the tester asks the subject to connect the numbers in order as quickly as possible. In Part B, numbers and letters are randomly distributed on an A4 paper and the tester asks the subject to connect alternating number and letter series as quickly as possible (e.g., 1, A, 2, B, 3, C). Lower score indicates better executive functions. Cangoz et al. (2007)^18^ published the normative data of the TMT in a sample of Turkish population.

### Statistical analyses

Data were analyzed with SPSS version 22.0 statistical software package program. Categorical variables were reported as frequency (percentage), and continuous variables were reported as mean ± SD. The results of Shapiro-Wilk test of data normality indicated the data were normally distributed. Differences in nominal variables between groups were analyzed using chi-square test. Initial values for continuous variables were compared between groups using independent- samples t-test. Initial and final values were compared within groups using paired-samples t test. Increment in MOCA total, attention, and delayed recall scores and decrement in TMT-A and TMT-B scores demonstrated a learning effect in the tests. Therefore, general linear model repeated measures was applied for the TMT-A, TMT-B, and MOCA total, attention, and delayed recall subtests. Two-factor repeated measure analysis of variance (ANOVA) was used to assess the effect of time × group (sleep deprivation) interaction. Results of Greenhouse-Geisser adjustment were reported if Mauchly’s test of sphericity was significant. Level of significance was .05.

## RESULTS

The study groups were comparable in terms of education level and mean typical sleep duration (*p*>.05 for both). The demographic characteristics of the groups are presented in Table 1. On the night of the study, mean sleep duration in the control group was 7.04±0.80 hours (range, 6-8 hours).

**Table 1.** Demographic characteristics of the participants.

	Sleep deprivation group		Control group		
	**n (21)**	**%**	**n (21)**	**%**	**p**
Education level					1
Primary school	0	0	0	0	
High school	21	100	21	100	
Bachelor’s degree	0	0	0	0	
Employment status					.54
Part-time	2	9.5	1	4.8	
Full-time	0	0	0	0	
Not working	19	90.5	20	95.2	
	**Mean±SD**		**Mean±SD**		**1**
**Age (years)**	21.76±2.04		21.76±2.04		
**Average sleep time (hours)**	7.71±0.85		7.25±0.68		.08

SD: Standard deviation.

The initial MOCA and TMT mean scores and standard deviation values of the sleep deprivation and control groups are shown in Table 2.

**Table 2.** Comparison of initial MOCA, TMT-A, and TMT-B scores between the sleep deprivation and control groups.

	Sleep deprivation	Control	p
	Mean±SD	Mean±SD	
**MOCA**	28.19±1.36	28.76±1.37	.184
Visuospatial	5.00±0.00	5.00±0.00	1
Naming	3.00±0.00	3.00±0.00	1
Attention	5.47±0.74	5.80±0.60	.120
Language	3.00±0.00	3.00±0.00	1
Abstraction	2.00±0.00	2.00±0.00	1
Delayed Recall	3.71±1.18	3.95±1.16	.515
Orientation	6.00±0.00	6.00±0.00	1
**TMT Part A**	19.50±4.61	18.39±4.36	.426
**TMT Part B**	51.96±13.15	45.27±27.50	.321

MOCA: Montreal cognitive assessment; TMT: Trail making test.

**Figure 1:**
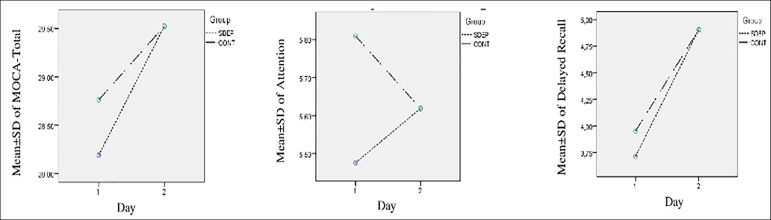
Comparisons of MOCA Total, Delayed Recall, and Attention Subtest Time X Group Interaction Between the Groups SDEP; SLEEP deprivation, CONT: Control group.

**Figure 2:**
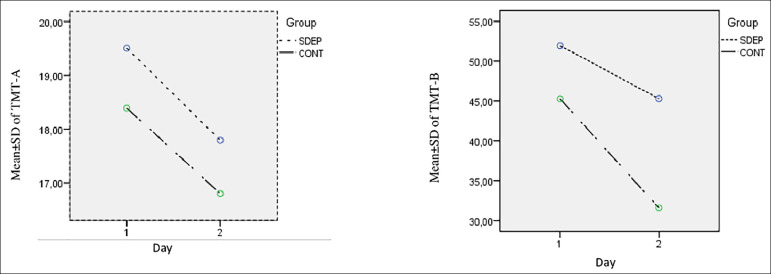
Comparisons of Trail Making Test A/B Time X group Interaction Between the groups.SDEO: Sleep depivation group, CONT: Control group.

No changes were observed between initial and final MOCA scores in the visuospatial, naming, language, abstraction, and orientation subtests in either group (*p*=1) (Tables 3 and 4). The general linear model repeated measures applied to TMT-A, TMT-B (Figure 1f), and MOCA total, attention, and delayed recall subtests (Figure 2) demonstrated insignificant effect of time × sleep deprivation interaction on the scores (*p*>.05).

## DISCUSSION

The main finding of this study is that cognitive functions assessed by MOCA and TMT remained unchanged after a single night of total sleep deprivation in healthy volunteers. This result may stem from two important factors. First, the cognitive test batteries used in the current study measure only short-term cognition. The second factor is that all participants in this study were young adults, who may be less affected by sleep deprivation.

Cognitive performance can be assessed under various conditions that may require sustained cognition or short-term cognition. MOCA and TMT are short tests that require short-term cognition, and one night of sleep deprivation may not be enough to impair short-term cognition. Bruin et al. (2017)^19^ cited cognitive task duration as a confounding factor in the assessment of cognition.

Sleep deprivation may affect different cognitive domains including executive functioning, working memory, episodic memory, and attention/alertness^12^. TMT mainly tests executive functioning, whereas MOCA evaluates naming, attention, memory, abstraction, language, and visuospatial functions and delayed recall skills. In the present study, we focused our analysis on the memory and attention domains of the MOCA because the sleep deprivation group showed no change in the other subtests.

There are numerous studies examining the effects of sleep deprivation or sleep restriction on executive functions in healthy individuals. Using TMT to assess executive function it was shown that four hours of partial sleep curtailment^13^ or four consecutive nights of sleep restriction^14^ had no effect on executive function in adolescents. Similarly, we failed to find any significant alteration in executive function after one-night sleep deprivation in young adults. Collectively, these results suggest that younger individuals may better tolerate short-term sleep deprivation. In contrast, middle-aged individuals showed a decrement in executive functions in a driving simulation after one-night sleep deprivation^20^.

Attention is the basis of cognitive functions. Reduced performance in attention tasks has been reported after 20 to 24 hours’ total sleep deprivation^21,22^. Contrary to the previous literature, we observed no change in attention in our sleep-deprived group. Several factors may affect susceptibility and/or resistance to the negative effects of sleep deprivation, including chronotype, age, sex, and habitual sleep length^12^. Although we controlled for age, sex, and average sleep length in our study, chronotype was not assessed.

Another domain of cognitive function is memory. There is no consensus in the literature regarding the causal relationship between sleep deprivation and memory alterations. Some studies provided evidence for the adverse effects of sleep deprivation on memory functions using the digit symbol substitution task, serial addition/subtraction task, N-back (0-, 1-, 2-, 3-back), continuous performance test^24,26^. However, other studies using the letter task, letter task plus, plus-L, and auditory 3-back task suggested that sleep deprivation did not affect memory functions^27,29^. In the current study, memory functions were unchanged following one night of sleep deprivation. To our knowledge, this is the first study to use MOCA to assess memory in sleep deprivation.

There are several limitations of this study that should be acknowledged. The most important limitation of the current study was the learning effect seen with the evaluation instruments, which may mask findings related to the cognitive functions. We performed the tests at two time points and expected to see some cognitive impairment after sleep deprivation. However, performance was better in each test at the second time point, independent of sleep status. These findings indicate that the participants learned the test conditions and exhibited better performance with repeated testing. Further studies should take the learning effect into consideration and utilize batteries which may exclude this factor. Second, we included only young adults in the study, which precludes the generalization of our findings to older populations. A third limitation was testing at 8 a.m.; measurements conducted later in the day may have revealed a decrement in cognitive test performance. Further studies should include repeated measures later in the day.

In conclusion, one night of sleep deprivation did not affect cognitive performance in the young adults in this study; however, the limited sample size requires caution when interpreting the study results. Cognitive functions are the basis of everyday functioning, including the coordination of activities of daily living, and disruptions in cognitive function have been shown to lead to injuries due to traffic and work-related accidents^30^. Therefore, it is an important and reassuring finding that a single night of sleep deprivation, which can be inevitable in modern society, caused no significant decrement in cognitive performance among young adults.
